# Comparison of image quality from filtered back projection, statistical iterative reconstruction, and model-based iterative reconstruction algorithms in abdominal computed tomography

**DOI:** 10.1097/MD.0000000000004456

**Published:** 2016-08-07

**Authors:** Yu Kuo, Yi-Yang Lin, Rheun-Chuan Lee, Chung-Jung Lin, Yi-You Chiou, Wan-Yuo Guo

**Affiliations:** a Department of radiology, Taipei Veterans General Hospital; b School of Medicine, National Yang-Ming University, Taipei, Taiwan, R.O.C.

**Keywords:** abdomen, computed tomography, image quality, iterative model reconstruction, iterative reconstruction, radiation dosage

## Abstract

The purpose of this study was to compare the image noise-reducing abilities of iterative model reconstruction (IMR) with those of traditional filtered back projection (FBP) and statistical iterative reconstruction (IR) in abdominal computed tomography (CT) images

This institutional review board-approved retrospective study enrolled 103 patients; informed consent was waived. Urinary bladder (n = 83) and renal cysts (n = 44) were used as targets for evaluating imaging quality. Raw data were retrospectively reconstructed using FBP, statistical IR, and IMR. Objective image noise and signal-to-noise ratio (SNR) were calculated and analyzed using one-way analysis of variance. Subjective image quality was evaluated and analyzed using Wilcoxon signed-rank test with Bonferroni correction.

Objective analysis revealed a reduction in image noise for statistical IR compared with that for FBP, with no significant differences in SNR. In the urinary bladder group, IMR achieved up to 53.7% noise reduction, demonstrating a superior performance to that of statistical IR. IMR also yielded a significantly superior SNR to that of statistical IR. Similar results were obtained in the cyst group. Subjective analysis revealed reduced image noise for IMR, without inferior margin delineation or diagnostic confidence.

IMR reduced noise and increased SNR to greater degrees than did FBP and statistical IR. Applying the IMR technique to abdominal CT imaging has potential for reducing the radiation dose without sacrificing imaging quality.

## 1 Introduction

Computed tomography (CT) of the abdomen is a crucial imaging technique for clinical diagnosis. The number of CT studies has been increasing since its inception; however, concerns regarding radiation risks caused by CT have also increased. CT may be responsible for 1.5% to 2% of all cancers in the United States.^[1]^ Several methods, such as tube current modulation, optimizing tube voltage, centering patients, tailoring scanning ranges and phases, and adjusting pitch, have been developed to reduce the radiation dose.^[2–4]^ New reconstruction algorithms, such as statistical iterative reconstruction (IR) and model-based iterative reconstruction (MBIR), produce higher imaging quality than does traditional standard filtered back projection (FBP) and also have high potential for reducing the radiation dose.^[5]^ IR algorithms render images through iterative cycles of estimation and correction based on different models.^[5]^ Statistical IR reduces image noise by employing models based on the photon statistics, removing random fluctuations in data measurements.^[6]^ In addition to statistical models, MBIR incorporates further models of system geometry and acquisition processes in the iterative cycles to fine-tune image quality.^[5]^

iDose4 (Philips Healthcare, Cleveland, OH) is a commercialized statistical IR that has a higher quality of chest–abdomen–pelvis CT images than that of FBP.^[7]^ Initial performance assessments have revealed that iterative model reconstruction (IMR) (Philips Healthcare) renders superior image quality.^[8,9]^ In iDose4, noise reduction is performed using statistical and structural models sequentially in the projection and image spaces.^[5]^ Noise can be further suppressed in IMR by incorporating additional system models into image processing.^[10,11]^

The present study objectively and subjectively compared the image quality in abdominal CT produced by IMR with iDose4 and FBP. Cystic structures in the abdomen were used to evaluate imaging quality; the regions of interest (ROIs) comprised distended urinary bladders and renal cysts. The urinary bladder contains homogeneous water-attenuated urine, which is often disturbed by the projection of streaky artifacts, caused by photon starvation and beam-hardening effects.^[12]^ Therefore, the urinary bladder is useful in evaluating image noise and the effectiveness of noise reduction. Simple renal cysts are homogeneous, spherical, and sharply demarcated, exhibiting a smooth border with a water attenuation of 0 to 20 Hounsfield units (HUs).^[13]^ Therefore, they are ideal targets for evaluating image noise.

## 2 Methods

### 2.1 Patient selection

This study was approved by our institutional review board (IRB). Written informed consent was waived by the IRB, given the retrospective nature of the study. During April 1 to September 30, 2015, 103 patients (60 males and 43 females, average age 64.8 ± 13.9 years) who participated in a clinically indicated contrast-enhanced abdominal CT study were retrospectively enrolled. Patients who satisfied the inclusion criteria were assigned into a urinary bladder group (45 males and 38 females, average age 62.1 ± 13.4 years) and a renal cyst group (30 males and 9 females, average age 74 ± 11.7 years). The inclusion criteria for the bladder group are as follows: distended urinary bladder and ability to draw a ROI size of >15 cm^2^ at least 0.5 cm away from the inner wall of the urinary bladder; no space-occupying lesions; no known history of urinary bladder operation; no placement of Foley tube or other medical devices; and no history of hematuria. The inclusion criteria for the cyst group are as follows: having a Bosniak category 1^[14]^ renal cyst >1 cm in diameter; no known kidney neoplasm; and no polycystic kidney disease. There were 5 patients having 2 renal cysts, which met the cyst group criteria.

### 2.2 Scanning techniques

Abdominal CT was performed from the lower thorax to the perineum in all patients. Images were obtained using a 256-slice multidetector unit (iCT; Philips Healthcare). All patients received 90 mL of iodinated contrast media (Ultravist 370; Bayer–Schering, Berlin, Germany) at 1.3 mL/s from a power injector. After a delay of 90 seconds following injection, abdominal CT scans were performed. The scan parameters were detector collimation of 64 × 0.625 mm, tube voltage of 120 kV, tube current employing the longitudinal dose modulation technique with an automatic current setting by analyzing the scout image, rotation time of 0.5 seconds, and pitch of 0.985. This protocol has been routinely employed in daily practice at our hospital.

### 2.3 Image reconstruction

All raw data were extracted and reconstructed through FBP, iDose4 (Level 4), and IMR (image definition of routine [IMR-R] and soft tissue [IMR-ST], noise reduction level from L1 to L3) by using a prereleased IMR prototype system. Data were reformatted for 5-mm slice thickness. Window settings were consistent in all reconstructed images, with a window level of 50 HU and window width of 400 HU.

### 2.4 Radiation dose

Mean tube current, CT dose index volume (CTDI_vol_) (measured in mGy), and dose-length products (DLPs) (measured in mGy-cm) were provided by the CT scanner. Effective radiation doses (measured in mSv) were calculated as the product of DLP and a conversion factor of 0.015 (mSv mGy^−1^ cm^−1^).^[15]^

### 2.5 Objective analysis

**Figure 1:**
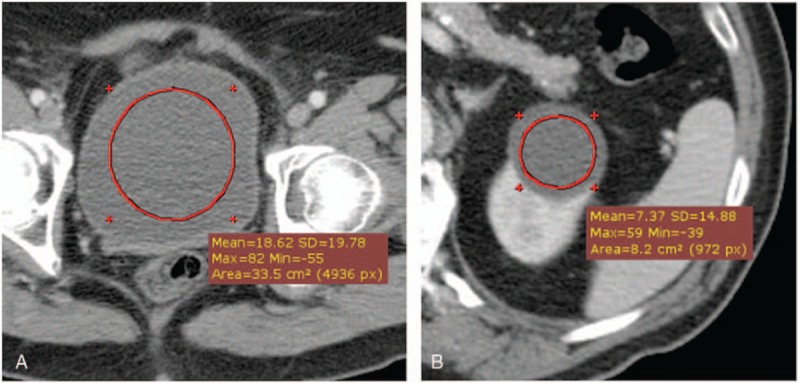
Examples of region of interest (ROI) drawings on the urinary bladder of a 65-year-old male patient (A) and on a renal cyst of an 88-year-old female patient (B). The requirements for drawing the ROIs on the urinary bladder comprised an ROI size of >15 cm^2^ and a border drawn at least 0.5 cm away from the inner wall of the urinary bladder. The border of the ROI drawn on the renal cysts was at least 0.2 cm away from the cyst walls. ROI = region of interest.

For each urinary bladder (n = 83) and renal cyst (n = 44), the ROIs were placed on all the reconstructed images at the same position by using the RadiAnt DICOM Viewer Version 1.9.16 (Medixant, Poland). The requirements for drawing the ROIs (average size 26.6 ± 9.1 cm^2^) on the urinary bladder comprised ROI size of >15 cm^2^ and a border drawn at least 0.5 cm away from the inner wall of the urinary bladder (Fig. 1A). The borders of the ROIs (average size 3.7 ± 3.2 cm^2^) drawn on the renal cysts were at least 0.2 cm away from the cyst walls (Fig. 1B). The ROIs were drawn and reviewed by researchers YK and YYL, respectively.

ROI measurements were recorded, comprising mean HU, standard deviation (SD), and maximum and minimal HUs. Image noise was defined as the SD of CT numbers in the ROI. The signal-to-noise ratio (SNR) was calculated as follows: 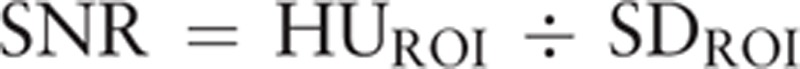


where HU_ROI_ is the mean CT number in the ROI and SD_ROI_ is the SD of the ROI.

### 2.6 Subjective analysis

Two radiologists specializing in abdominal radiology (with 30 and 6 years of professional experiences, respectively) evaluated the image quality. The radiologists were blinded for the types of reconstruction algorithms and patient clinical information. The radiologists evaluated 80 randomly sorted images from the urinary bladder and cyst groups on a picture archiving and communicating system workstation. The image dataset consisted of 4 selected reconstruction algorithms (i.e., FBP, iDose4, IMR-R-L1, and IMR-ST-L1) from 20 randomly selected patients with a window level of 50 HU and window width of 400 HU. Subjective image quality was scored using a 4-point Likert scale (1, = strongly agree; 4 = strongly disagree). For the bladder group, the radiologists were asked whether the overall image quality was excellent, whether there was no image noise, and whether there was no blotchy appearance. For the cyst group, in addition to the aforementioned 3 questions, the radiologists evaluated whether the margin was well demarcated and whether they were highly confident regarding their diagnoses.

### 2.7 Statistical analysis

All statistical analyses were performed using SPSS Version 22.0 software (SPSS Inc, Chicago, IL), and the results were expressed as mean ± SD. *P* ≤ 0.05 was considered significant. One-way analysis of variance (ANOVA) was applied for objective analysis and comparing the mean value, objective image noise, and SNR. The Levene test demonstrated inhomogeneity in the objective image noise and SNR datasets. Subsequently, the Welch test and the Games–Howell posthoc test were employed for statistical analysis of these 2 datasets. The independent sample *t* test was used to investigate any significant difference in image noise and SNR between the groups for each reconstruction algorithm.

For subjective analysis, the Friedman test was performed, with the Bonferroni-corrected Wilcoxon-signed rank test employed for posthoc analysis. Performance of the 4 reconstruction algorithms was subjectively graded. The significance threshold for the posthoc analysis was 0.0083 (0.05 divided by 6). Cohen κ test was applied for evaluating the interobserver agreement between the 2 radiologists. The results were interpreted as follows: κ ≤0, no agreement; κ = 0.01–0.20, non-to-slight agreement; κ = 0.21–0.40, fair agreement; κ = 0.41–0.60, moderate agreement; κ = 0.61–0.80, substantial agreement; and; κ = 0.81–1.00, near-perfect agreement.^[16]^

## 3 Results

### 3.1 Radiation dose

The average mean tube current was 160 ± 58.9 mA. The average CTDI_vol_ was 11.2 ± 4.1 mGy. The average DLP was 579.1 ± 242.3 mGy-cm. The average effective radiation dose was 8.7 ± 3.6 mSv.

### 3.2 Objective image quality assessment

Mean CT number, image noise, and SNR of the bladder and cyst groups are summarized in Table 1. No significant difference in the mean CT number between each reconstruction method was observed in either the bladder group (*P* = 0.97) or the cyst group (*P* = 0.86), as determined through one-way analysis of variance. The significance values of image noise and SNR for the bladder and cyst groups are listed in Tables 2 and 3, respectively.

**Table 1:**
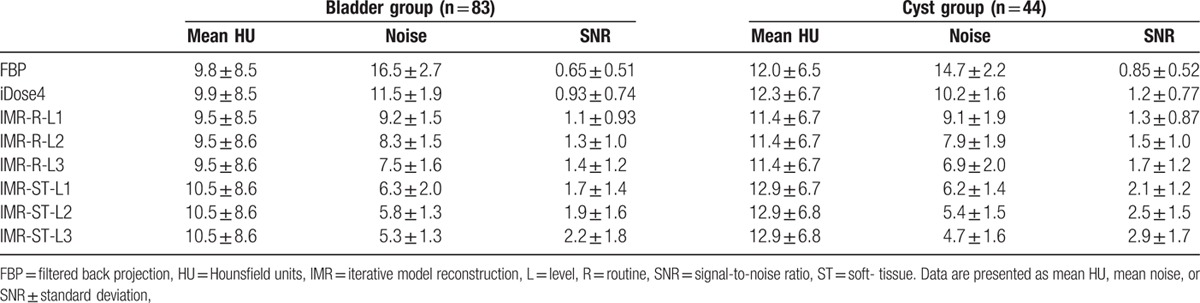
Mean CT number in Hounsfield units, noise, and signal-to-noise ratio for the bladder group and the cyst group.

**Table 2:**
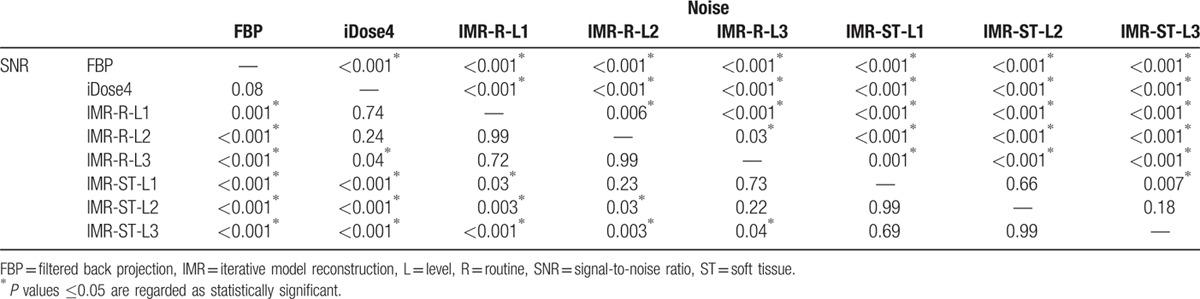
*P* values of image noise and signal-to-noise ratio for the bladder group—comparisons between FBP, iDose4, IMR-R-L1, IMR-R-L2, IMR-R-L3, IMR-ST-L1, IMR-ST-L2, and IMR-ST-L3 by one-way analysis of variance with Games–Howell post-hoc test.

**Table 3:**
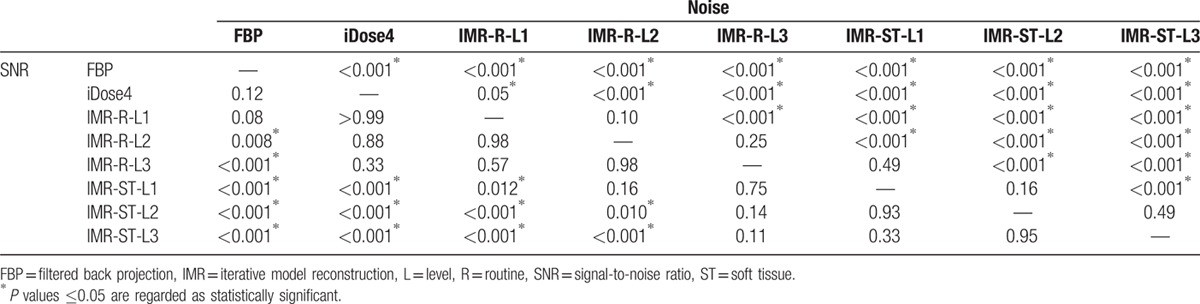
*P* values of image noise and signal-to-noise ratio for the cyst group—comparisons between FBP, iDose4, IMR-R-L1, IMR-R-L2, IMR-R-L3, IMR-ST-L1, IMR-ST-L2, and IMR-ST-L3 by 1-way analysis of variance with Games–Howell post-hoc test.

**Figure 2:**
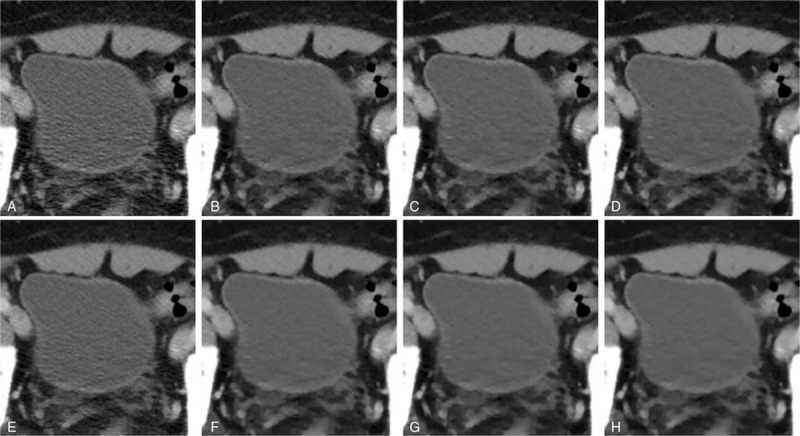
Comparison of abdominal computed tomography image quality focusing on the urinary bladder of a 63-year-old male patient. (A) FBP; (B) IMR-R-L1; (C) IMR-R-L2; (D) IMR-R-L3; (E) iDose4; (F) IMR-ST-L1; (G) IMR-ST-L2; and (H) IMR-ST-L3. Noise granules were evident in the FBP (A) and iDose4 (E) reconstruction algorithms. Noise was reduced after applying IMR (B–H), yielding images that were more homogenous. FBP = filtered back projection, IMR = iterative model reconstruction, L = level, R = routine, ST = soft tissue.

For the bladder group (Fig. 2), FBP demonstrated the highest mean image noise (16.5 ± 2.7), whereas IMR-ST-L3 demonstrated the lowest mean image noise (5.3 ± 1.3). FBP image noise was significantly (*P* < 0.001) higher than that of iDose4 (11.5 ± 1.9) and all IMR algorithms (*P* < 0.001). Furthermore, the image noise of iDose4 was significantly higher than that of all IMR algorithms (*P* < 0.001). iDose4, IMR-R-L1 (9.2 ± 1.5), and IMR-ST-L3 (5.3 ± 1.3) demonstrated 30.5%, 44.5%, and 67.8% noise reduction, respectively, evidencing higher performance than that of the FBP algorithm. IMR-R-L1 and IMR-ST-L3 respectively achieved 20.1% and 53.7% noise reduction, evidencing higher performance than that of iDose4. All IMR-R algorithms demonstrated a significantly (*P* ≤ 0.001) higher image noise than did the IMR-ST group. However, there was no significant difference in image noise between IMR-ST-L1 and IMR-ST-L2 or between IMR-ST-L2 and IMR-ST-L3.

All IMR algorithms demonstrated a significantly higher SNR than did FBP (0.65 ± 0.51, *P* ≤ 0.001). All IMR-ST algorithms and IMR-R-L3 (1.3 ± 1.0) demonstrated significantly (*P* < 0.05) higher SNR than did iDose4 (0.93 ± 0.74) and FBP. No significant differences in SNR were observed among the IMR-R or IMR-ST algorithms.

**Figure 3:**
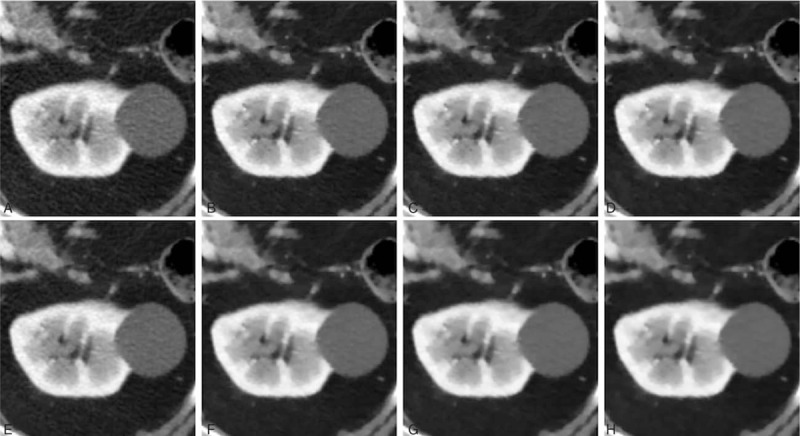
Comparison of abdominal computed tomography image quality focusing on a renal cyst in an 83-year-old male patient. (A) FBP; (B) IMR-R-L1; (C) IMR-R-L2; (D) IMR-R-L3; (E) iDose4; (F) IMR-ST-L1; (G) IMR-ST-L2; and (H) IMR-ST-L3. The 3.4-cm renal cyst appeared inhomogeneous in the FBP (A) and iDose4 (E) reconstruction algorithms. Noise was reduced after applying IMR (B–H), yielding smoother images. FBP = filtered back projection, IMR = iterative model reconstruction, L = level, R = routine, ST = soft tissue.

In the cyst group (Fig. 3), FBP demonstrated the highest mean image noise (14.7 ± 2.2), whereas IMR-ST-L3 demonstrated the lowest mean image noise (4.7 ± 1.6). FBP image noise was significantly (*P* < 0.001) higher than that of iDose4 (10.2 ± 1.6) and all IMR algorithms (*P* < 0.001). Similar to the bladder group, iDose4 image noise was significantly higher than that of all IMR algorithms (*P* ≤ 0.05). iDose4, IMR-R-L1 (9.1 ± 1.9), and IMR-ST-L3 (4.7 ± 1.6) respectively demonstrated 30.2%, 38.1%, and 67.7% noise reduction, evidencing higher performance than that of the FBP algorithm. IMR-R-L1 and IMR-ST-L3 achieved noise reductions of 11.3% and 53.7%, respectively, evidencing higher performance than that of iDose4. All IMR-R algorithms achieved a significantly (*P* < 0.001) higher image noise than the IMR-ST group did, with no significant difference (*P* = 0.49) between IMR-R-L3 (6.9 ± 2.0) and IMR-ST-L1 (6.2 ± 1.4). There was no significant difference in image noise between IMR-ST-L1 and IMR-ST-L2 or between IMR-ST-L2 and IMR-ST-L3.

IMR-ST-L3 (2.9 ± 1.7) demonstrated the highest SNR among all algorithms. FBP (0.85 ± 0.52) demonstrated the lowest SNR with no significant difference compared with either iDose4 (*P* = 0.12) or IMR-R-L1 (*P* = 0.08). No significant difference in image noise was observed among the IMR-R or IMR-ST algorithms.

**Figure 4:**
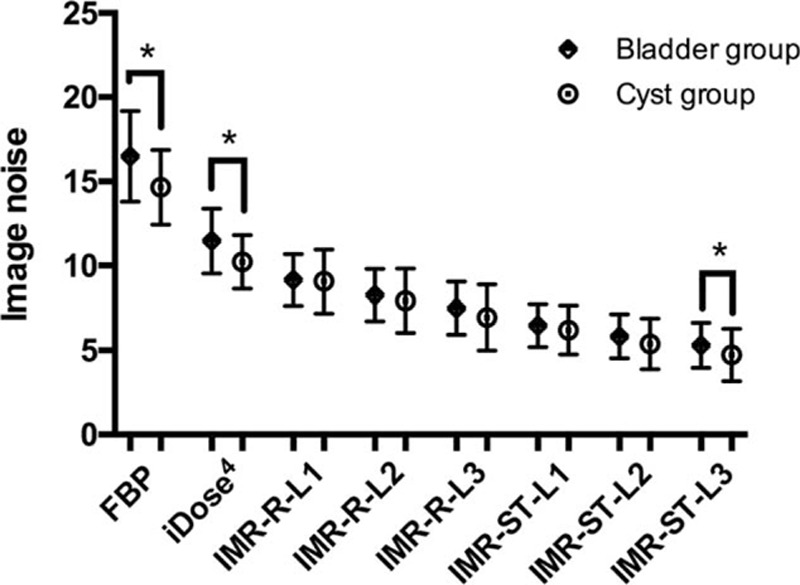
Image noise in the bladder and cyst groups were paired and compared for each reconstruction algorithm. Image noise of the bladder group under FBP, iDose4, and IMR-ST-L3 reconstruction algorithms was significantly (∗*P* < 0.05) higher than that of the cyst group. FBP = filtered back projection, IMR = iterative model reconstruction.

**Figure 5:**
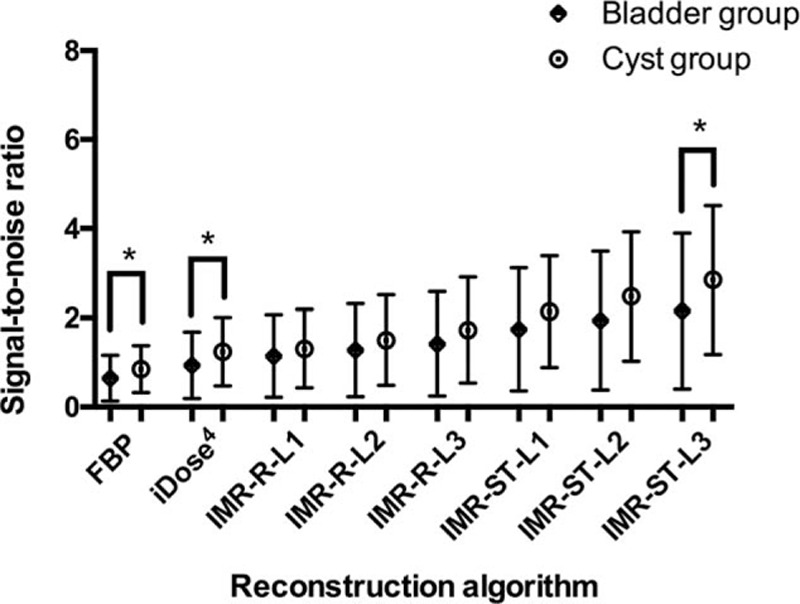
Signal-to-noise ratio in the bladder and cyst groups were paired and compared respectively for each reconstruction algorithm. The signal-to-noise ratio of the bladder group under FBP, iDose4, and IMR-ST-L3 reconstruction algorithms was significantly (∗*P* < 0.05) lower than that of the cyst group. FBP = filtered back projection, IMR = iterative model reconstruction.

Image noise of the bladder group under FBP, iDose4, and IMR-ST-L3 reconstruction algorithms was significantly (*P* < 0.05) higher than that of the cyst group (Fig. 4). Similarly, the SNR of the bladder group under FBP, iDose4, and IMR-ST-L3 reconstruction algorithms was significantly (*P* < 0.05) lower than that of the cyst group (Fig. 5). No significant difference was observed in image noise or SNR between the bladder group and the cyst group for IMR-R-L1, IMR-R-L2, IMR-R-L3, IMR-ST-L1, and IMR-ST-L2.

### 3.3 Subjective image quality assessment

The subjective image-quality scores of the bladder and cyst groups are summarized in Tables 4 and 5.

**Table 4:**
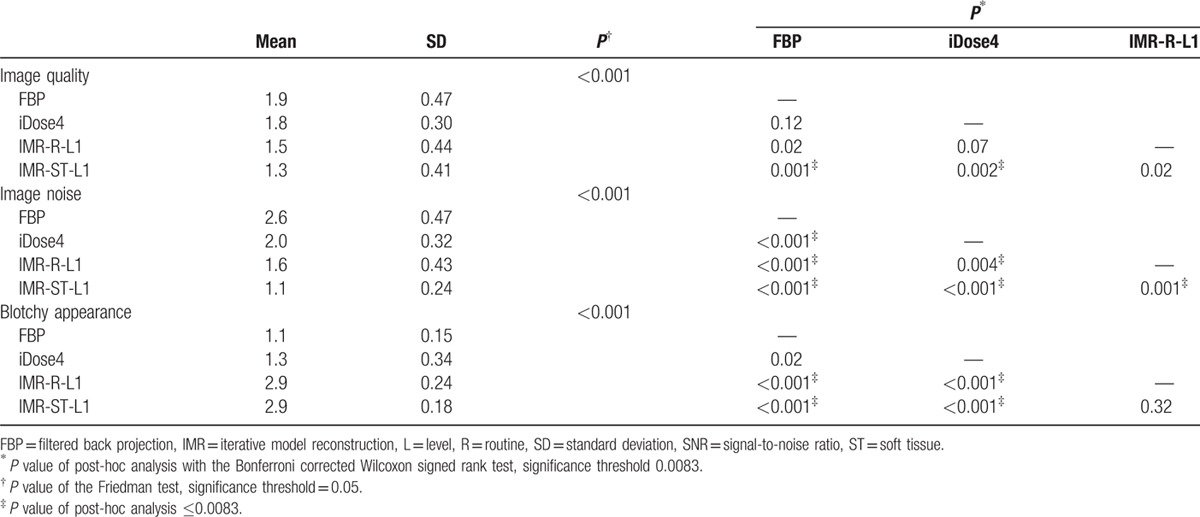
Summary of subjective scores of the bladder group.

**Table 5:**
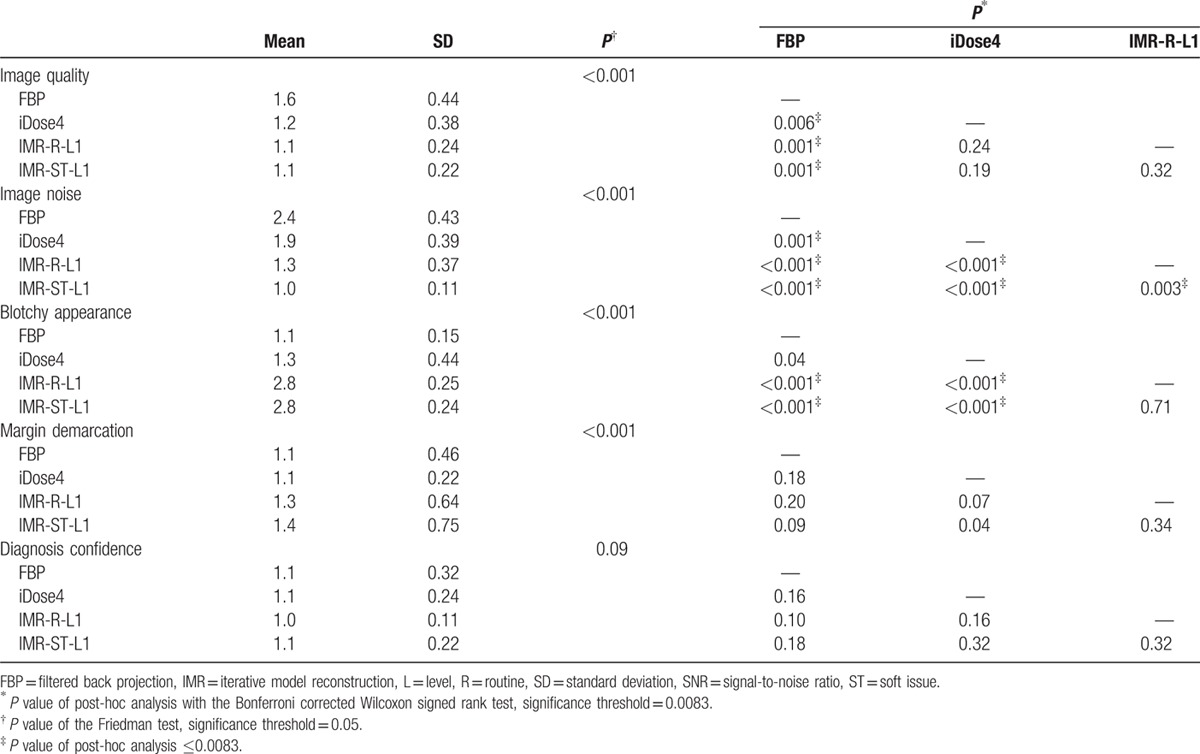
Summary of subjective scores of the cyst group.

In the bladder group, the subjective image quality for IMR-ST-L1 was significantly higher than that for FBP and iDose4 reconstruction algorithms. FBP and IMR-ST-L1 demonstrated the highest and lowest noise image appearance, respectively. In addition, the IMR reconstructions (i.e., IMR-R-L1 and IMR-ST-L1) demonstrated a more prominent blotchy appearance than did the non-IMR reconstruction methods (i.e., FBP and iDose4).

For the cyst group, FBP achieved the poorest subjective image quality. FBP demonstrated the highest noise image appearance, whereas IMR-ST-L1 demonstrated the lowest noise image appearance. The IMR reconstructions (i.e., IMR-R-L1 and IMR-ST-L1) demonstrated a more prominent blotchy appearance than did the non-IMR reconstruction methods (i.e., FBP and iDose4). No difference was observed among the 4 reconstruction methods regarding either margin demarcation or diagnosis confidence in the cyst group.

The interobserver agreements between the 2 radiologists varied from moderate to substantial (κ = 0.54–0.78, *P* < 0.001) and are listed in Table 6.

**Table 6:**
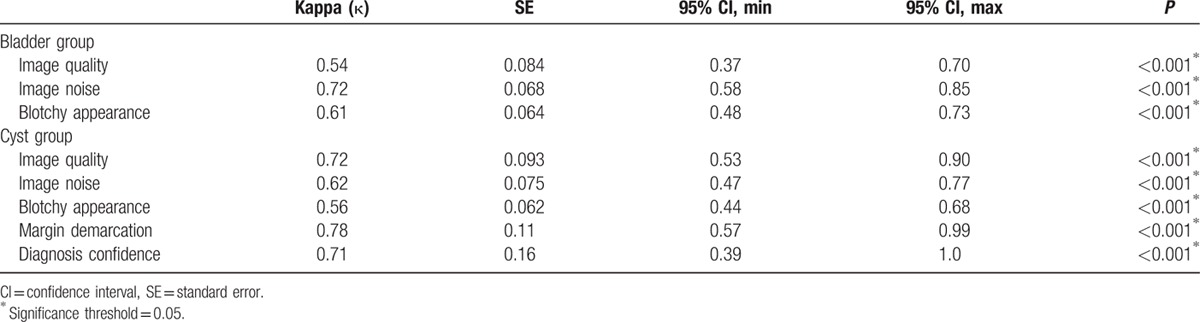
Cohen κ test for interobserver agreement.

## 4 Discussion

IMR is a newly developed MBIR. Only a few in vivo studies have validated the application of IMR in abdominal CT.^[8,17,18]^ In this study, the image quality among traditional FBP, iDose4, and IMR reconstruction algorithms in the abdominal CT study was evaluated by analyzing image noise, SNR, and subjective grading. The ROIs were placed on reconstructed images of the urinary bladder and renal cysts. The homogenous and nonenhancing qualities of these 2 cystic structures make them ideal anatomical locations for evaluating image noise and quality. Furthermore, our literature review indicated that this is the first study to evaluate the performance of the IR algorithm by analyzing the urinary bladder and renal cysts.

Suzuki et al^[8]^ reported that IMR has a superior image quality to that of iDose4, both objectively and subjectively, in patients with chronic liver diseases, reporting a 40.2% reduction in image noise of the liver parenchyma with iDose4 compared with that of FBP, as well as a 55.8% reduction in image noise with IMR, compared with that of iDose4. Our objective evaluation of the bladder and cyst groups revealed that iDose4 typically produces a less noisy image and superior SNR than does FBP, with a 30.2% reduction in image noise. IMR is superior to iDose4, demonstrating noise reduction of 11.3% to 53.7% (Tables 2 and 3). Applying higher levels of noise reduction and soft tissue definition in the IMR algorithms yielded superior results. Subjective grading of image noise was consistent with the objective results, with IMR-ST-L1 demonstrating the highest performance, followed by IMR-R-L1, iDose4, and FBP (Tables 4 and 5). The objective SNR assessment had the same ranking order as that of the objective image noise assessment. In both the bladder and cyst groups, IMR-ST-L3 yielded the highest SNR. The results were predictable because IMR employed a high number of models for iterative correction cycles.^[8]^

The differences in SNR values among the reconstruction algorithms in both groups were not as significant as the differences in image noise (Tables 2 and 3). For example, significant differences in image noise of renal cysts among the IMR-R-1, iDose4, and FBP reconstruction algorithms were observed with no significance regarding SNR. This finding is consistent with those of previous studies. Khawaja et al^[17]^ calculated SNR by dividing the mean HU of the ROI by the background noise, discovering no significant difference between the FBP and IMR reconstructions in ultralow-dose abdominal CT. In subjective image noise assessment, IMR-ST-L1 outperformed IMR-R-L1, iDose4, and FBP in both the bladder and cysts group. This finding is consistent with that of Park et al's,^[19]^ who reported that IMR images are subjectively less noisy than are images from both iDose4 and FBP reconstruction algorithms, even at ultralow doses.

Theoretically, the urinary bladders had a greater image noise and lower SNR than did the renal cysts because the urinary bladder is often the projection site of streaky artifacts caused by photon starvation and beam-hardening effects in the pelvic cavity^[12]^; our findings are consistent with this suggestion. However, significance could only be observed for the FBP, iDose4, and IMR-ST-L3 reconstruction algorithms (Figs. 4 and 5). This can be partly explained by the ability of IMR algorithms to eliminate the streaky artifacts in the bladder group to a certain degree. Subsequently, the differences of image noise and SNR between the bladder and cyst groups became less significant. However, this cannot fully explain the unexpected significant differences in both image noise and SNR between the bladder and cyst groups for the IMR-ST-L3 reconstruction algorithm.

Blotchy appearance is a common problem in MBIRs from various manufacturers,^[9]^ raising concerns about their potential adverse effects on lesion detection. Suzuki et al^[8]^ argued that no significant blotchy appearance was observed on IMR images compared with either iDose4 or FBP algorithms. However, subjective grading in our study revealed that IMR rendered significantly blotchier images than did both FBP and iDose4. iDose4 did not yield a significantly blotchier image appearance than did FBP. For IMR, the soft tissue image definition was not significantly blotchier than was the routine image definition (*P* < 0.05 in both bladder group and cyst group). No in vivo studies have clarified whether a blotchy appearance dampens the diagnostic accuracy of the abdominal CT image for IMR. In our study, despite the significantly blotchier appearance of IMR images compared with those of iDose4 and FBP, no significant negative effect on the margin demarcation or diagnostic confidence was observed in the cyst group. Volders et al^[20]^ reported that Veo (GE Healthcare, Milwaukee, WI), an MBIR algorithm, has a superior detection ability to that of an adaptive statistical IR algorithm (ASIR; GE Healthcare), with a reduced radiation dose for small liver metastases (<10 mm). Shuman et al^[21]^ reported that all clinically reportable findings identified in statistical IR algorithm images employing a standard radiation dose (ASIR; GE Healthcare) were all identified in MBIR algorithm (Veo; GE Healthcare) images with 60% reductions in the radiation dose. Although current evidence indicates the noninferior lesion-detection ability of MBIR, its gradual introduction in clinical practice is required for radiologists and clinicians to adapt to and have confidence in the new appearance of CT images.^[9]^

Studies have reported that iDose4 can considerably reduce the radiation dose while maintaining acceptable image quality. By applying the iDose4 protocol in chest–abdomen–pelvis CT examinations, Arapakis et al^[7]^ achieved a 46.5% reduction in the radiation dose, exhibiting a higher performance than that of the FBP protocol. Veldhoen et al^[22]^ reported that when evaluating urolithiasis, a 50% reduction in the radiation dose for the iDose4 protocol yielded an image quality comparable to that of the FBP protocol. Our study revealed that the image quality rendered by the IMR reconstruction algorithm was even higher than that achieved by iDose4, both objectively and subjectively, suggesting that IMR may have potential for reducing the radiation dose to a greater degree than does iDose4. Khawaja et al^[17]^ reported similar lesion-detection rates between a standard-dose FBP reconstruction and 85% dose-reduced IMR reconstruction in abdominal CT studies of smaller-sized patients. Extreme reduction in the radiation dose may result in unwanted artifacts and potential false-positive or false-negative diagnoses. Park et al^[19]^ indicated that for evaluating urolithiasis ≥3 mm, an ultralow-dose (0.68 mSv) CT image produced by IMR algorithms had a comparable diagnostic ability to that of the regular dose (8.3 mSv) scans reconstructed by a FBP algorithm. Additional studies are required to determine a balance between the radiation dose and image quality without compromising the diagnostic ability or accuracy.

This study has several limitations. First, only cystic structures were analyzed in this study (urinary bladder and renal cyst). An abdominal CT study comprises various structures with diverse tissue densities and properties; the findings of our study may not be generalizable. Second, although the diagnosis of simple renal cysts could be supported by other image modalities (e.g., ultrasonography or MRI) in most of the cases (33/39 patients), no pathological examination was performed. Third, no reduced radiation scan was performed in this study. Forth, the reconstruction system was based on a prereleased prototype IMR. Therefore, this study may not demonstrate the complete strength of IMR.

To summarize, the IMR reconstruction algorithm significantly improved image quality both objectively and subjectively, achieving 11.3% to 53.7% noise reduction, exhibiting a higher performance than that of its predecessor, iDose4. The findings herein suggest potential for reducing the radiation dose by applying IMR to abdominal CT images.
