# Comparison of image quality from filtered back projection, statistical iterative reconstruction, and model-based iterative reconstruction algorithms in abdominal computed tomography: Erratum

**DOI:** 10.1097/MD.0000000000005663

**Published:** 2016-12-09

In the article “Comparison of image quality from filtered back projection, statistical iterative reconstruction, and model-based iterative reconstruction algorithms in abdominal computed tomography”,^[1]^ which appeared in Volume 95, Issue 31 of *Medicine*, funding information was originally omitted. The funding statement should read as follows: This study was supported by grants from the Center of Excellence for Cancer Research at Taipei Veterans General Hospital (MOHW104-TDU-B-211-124-001), Taiwan, from Taipei Veterans General Hospital (V105C-029), Taipei, Taiwan.
